# Polymorphisms in Survivin (*BIRC5* Gene) Are Associated with Age of Onset in Breast Cancer Patients

**DOI:** 10.1155/2019/3483192

**Published:** 2019-07-28

**Authors:** Ilona Sušac, Petar Ozretić, Maja Gregorić, Mirela Levačić Cvok, Maja Sabol, Sonja Levanat, Diana Trnski, Domagoj Eljuga, Sven Seiwerth, Gorana Aralica, Mladen Stanec, Vesna Musani

**Affiliations:** ^1^Eljuga Polyclinic, 10000 Zagreb, Croatia; ^2^Division of Molecular Medicine, Ruđer Bošković Institute, 10000 Zagreb, Croatia; ^3^Zagreb Health School, 10000 Zagreb, Croatia; ^4^Kardinal Alojzije Stepinac Krašić Primary School, 10454 Krašić, Croatia; ^5^Department for Oncoplastic and Reconstructive Surgery, University Hospital for Tumors, 10000 Zagreb, Croatia; ^6^Institute of Pathology, University of Zagreb School of Medicine, 10000 Zagreb, Croatia; ^7^Clinical Department of Pathology and Cytology, University Hospital Centre Zagreb, 10000 Zagreb, Croatia; ^8^Department of Pathology, Clinical Hospital Dubrava, 10000 Zagreb, Croatia

## Abstract

Survivin, encoded by* BIRC5* gene (baculoviral IAP repeat containing 5), belongs to the family of inhibitors of apoptosis proteins (IAPs). In mammalian cells it participates in the control of mitosis, apoptosis regulation, and cellular stress response. Its expression is increased in almost all types of cancers. The aim of this study was to investigate the role of* BIRC5* polymorphisms in breast cancer (BC) and to connect survivin expression with various clinicopathological characteristics of BC patients. Blood and archival tumour tissue samples were collected from 26 BC patients from Croatia. Survivin expression was determined immunohistochemically.* BIRC5* promoter, coding region, and 3'UTR were genotyped. DNA from 74 healthy women was used as control.* BIRC5* polymorphisms and survivin expression were tested against age of onset, histological grade, tumour type and size, lymph node status, oestrogen, progesterone, Her2, and Ki67 status. Numbers of samples with weak, moderate, and strong survivin expression were 9 (33.3%), 11 (40.7%), and 7 (25.9%), respectively. Most patients had nuclear survivin staining (92.6%). High survivin expression was significantly associated with negative oestrogen receptor status (p=0.007) and positive Ki67 expression (p=0.032). Ki67 expression was also positively correlated with histological grade (p=0.0009). Fourteen polymorphisms were found in BC samples, located mostly in promoter and 3'UTR of* BIRC5*. There was no significant difference in the distribution of polymorphisms between BC and control samples. Among clinicopathological characteristics of BC patients, alleles of five* BIRC5* polymorphisms were associated with younger age of onset: c.-644T>C (55.8 years [y] vs. 48.1 y; p=0.006), c.-241C>T (54.2 y vs. 45.0; p=0.029), c.9809T>C (55.8 y vs. 48.1 y; p=0.006), c.-1547C>T (58.3 y vs. 50.9 y; p=0.011), and c.9386T>C (50.8 y vs. 59.5 y; p=0.004). To assess the significance of* BIRC5* polymorphisms and survivin expression as predictive and prognostic biomarkers for BC further research with a larger sample size is needed.

## 1. Introduction

Breast cancer (BC) is one of the most common malignancies among women worldwide. Despite generally good prognosis for BC patients, there is a wide variation in survival [1]. Some of the common risk factors for BC include age, genetic background, hormonal factors, reproductive and menstrual history, excessive alcohol consumption, radiation, benign breast disease, and obesity [2]. Genetic variation has been shown to affect both susceptibility and prognosis of BC [1]. Genome wide association studies (GWAS) have recently been used to identify new loci for BC, and so far almost 100 loci have been identified [2]. In addition to providing new prognostic markers, genetic determinants of prognosis can also give new biological insight for progression of BC [3]. Survivin, encoded by* BIRC5* (baculoviral IAP repeat containing 5) gene, belongs to the family of inhibitors of apoptosis (IAP) proteins [4]. It is the smallest of eight IAP proteins and contains a single Baculovirus IAP Repeat (BIR), which is the hallmark of these molecules [5, 6]. Survivin is a multifunctional protein and in mammalian cells it participates in at least three cellular processes: the control of mitosis, the regulation of apoptosis, and the cellular stress response [5]. Survivin is strongly expressed during development, while in healthy organisms it is not expressed in differentiated tissues, and its expression is markedly increased in almost all types of cancers (including bladder cancer, lung cancer, breast cancer, stomach, oesophagus, pancreas, liver, uterine, ovarian, and haematological cancers) [7]. Elevated expression of survivin in BC is associated with increased resistance to chemo- and radiotherapy, increased grade, lymph node invasion, and tumour size, as well as decreased survival rate [8]. According to Marsicano et al., breast cancer patients with higher expression of* BIRC5* have significantly greater chance of developing metastasis than patients with lower expression [9]. Survivin is expressed in a cell cycle-regulated manner, being maximally expressed during the G2/M phase, followed by rapid decline of both the mRNA and protein levels at the G1 phase [10]. Several* BIRC5* polymorphisms were shown to be associated with susceptibility (gastric [11], bladder [12], and hepatocellular [13]), survival (colorectal [14] and breast [15]), or age of onset (ovarian cancer [4]). Some of these polymorphisms create binding sites for either transcription factors or regulatory miRNA and potentially can influence survivin levels. Survivin has been proposed as a potential target for anticancer intervention and several survivin inhibitors and survivin-related molecular therapies are in development [16]. The purpose of this preliminary study was to investigate the role of* BIRC5* polymorphisms in BC. The connection between polymorphisms found and protein expression in tumour tissue and their association was also assessed.

## 2. Materials and Methods

### 2.1. Study Subjects

This study included BC patients who had their regular check-up at the Eljuga Polyclinic and agreed to participate. Upon signing the consent forms their blood was drawn. 26 patients, diagnosed between 2000 and 2012 and whose archival paraffin embedded tumour tissue was available from archive at the University Hospital for Tumours, University Hospital Centre “Sestre Milosrdnice,” were included in the study. One patient had a bilateral BC and both tumours were available for testing. Demographic and clinicopathological data on patients was collected from medical records. Demographic characteristics included age of onset and time since operation, and clinicopathological characteristics included histological grade, tumour size, oestrogen and progesterone receptor status, Her2 status, lymph node status, type, and Ki67 status (determined routinely as part of the diagnostic procedure in the clinic). Two 5-*μ*m sections of tumour tissue were cut from paraffin block for immunohistochemical analysis. DNA samples from 74 healthy women with no history of BC (median age 80, range 65-101) were collected in our previous study [17]. Although usual case-control studies are age matched, in this case it was more appropriate to use elderly healthy population as control. This population is less likely to have cancer predisposing polymorphisms. The study was approved by the Institutional Ethics Committee (EP-6820/13-11).

### 2.2. Immunohistochemical Staining

Survivin protein was analysed as follows: slides were deparaffinized, prewarmed in Epitope Retrieval Solution (Dako, Glostrup, Denmark) and kept at 95-99°C for 20 min, left to cool for additional 20 min, and then washed in PBS 3x5 min. Endogenous peroxidase was blocked with 3 % H_2_O_2_/methanol for 10 min and then washed 3x5 min in PBS. The sample was circled by PAP-PEN (Kiyota, Baltimore, MD). Protein block serum-free (DAKO) was added for 10 min.

Immunohistochemical staining was performed using monoclonal rabbit anti-survivin antibody (1:200, 71G4B7, Cell Signalling Technology, Danvers, MA). Following an overnight incubation, the immunodetection was completed using the LSAB Visualization System (DAKO) utilizing 3, 3-diaminobenzidine (DAB) chromogen as substrate, according to the manufacturer's instructions. All sections were counterstained with haematoxylin (DAKO). Negative controls were obtained by omitting the primary antibody.

Stained slides were examined by two experienced pathologists. Samples were scored between 1 and 3 based on survivin expression, taking into account the percentage of positive cells and the staining intensity, as previously described by Xu et al. [18]. Staining intensity was expressed as + (weak), ++ (moderate), and +++ (intense). Percentage of tumor cells positive for survivin staining was categorized as 0 (no positive cells), 1 (<10 %), 2 (10-50 %), and 3 (>50 %). Final score was then generated by multiplying the staining intensity with the percentage of positive cells and ranged from 0 (no stained tumor cells) to 9 (>50 % of intensively stained tumor cells). The final score of 0 was considered as negative (0), 1-3 as weak (1), 4-6 as moderate (2), and 7-9 points as strong (3).

### 2.3. SNP Selection and Genotyping

Whole coding region of the* BIRC5* gene (including alternative exons 2alpha, 2B, and 3B) was genotyped, including the most common SNPs in* BIRC5* promoter and the 3' untranslated region (3'UTR). Polymorphisms that were selected from the National Center for Biotechnology Information SNP database (http://www.ncbi.nlm.nih.gov/snp) are those usually used in association studies (see [4, 10, 13–15, 19–21]). The majority of these studies have found that polymorphisms in 5' and 3' regions have the greatest impact on survivin expression [22] and might contribute to risk of various cancers [21]. Genomic DNA from blood was extracted by the salting out method. Thirteen PCR fragments were analysed using high resolution melting analysis on High-Resolution Melter (HR-1, Idaho Technology, USA) as described by Cvok et al. [17] followed by Sanger sequencing (ABI PRISM 310 Genetic Analyzer, Applied Biosystems, USA). Due to presence of 5 different polymorphisms in the PCR product of exon 4, it was directly sequenced. PCR primer sequences and cycling conditions are listed in Supplementary Table 1.

### 2.4. Statistical Analysis

Genotype and allele frequencies of patients and controls were compared using *χ*2 test. Deviation from the Hardy–Weinberg equilibrium (HWE) and linkage disequilibrium were calculated using the online tool SHEsis [http://analysis.bio-x.cn/myAnalysis.php] [23]. The *χ*2 test was used for testing the association between genetic data and categorical clinicopathological variables. Independent samples t-test and one-way analysis of variance with Tukey-Kramer post hoc test were used for testing the difference between continuous (age of onset and logarithmic transformed tumour size) and categorical variables. Two-tailed p-values less than 0.05 were considered statistically significant. Statistical analysis was performed using MedCalc v18.2.1 (MedCalc Software bvba).

## 3. Results and Discussion

### 3.1. Patient Characteristics

A total of 26 patients with BC were recruited in this study. The main clinical and histopathological features are summarized in Table 1. The median age of patients at diagnosis was 53 years (range 37-69): only one patient (3.8%) was younger than 40 years, 34.6% were between 40 and 50 years, 30.8% were between 50 and 60, and 30.8% were older than 60. The majority of patients (96.2%) were still alive at the end of the study so survival analysis was not performed.

Tumour size was associated with positive lymph nodes (p=0.004). Histological grade was associated with Ki67 expression (p=0.0009). All samples with histological grade 1 were negative for Ki67, while only one sample with histological grade 3 was negative for Ki67. Progesterone receptor (PR) and oestrogen receptor (ER) status were weakly positively associated (0.053). These results are in agreement with previously published international data [24–27].

### 3.2. Survivin Expression

**Figure 1:**
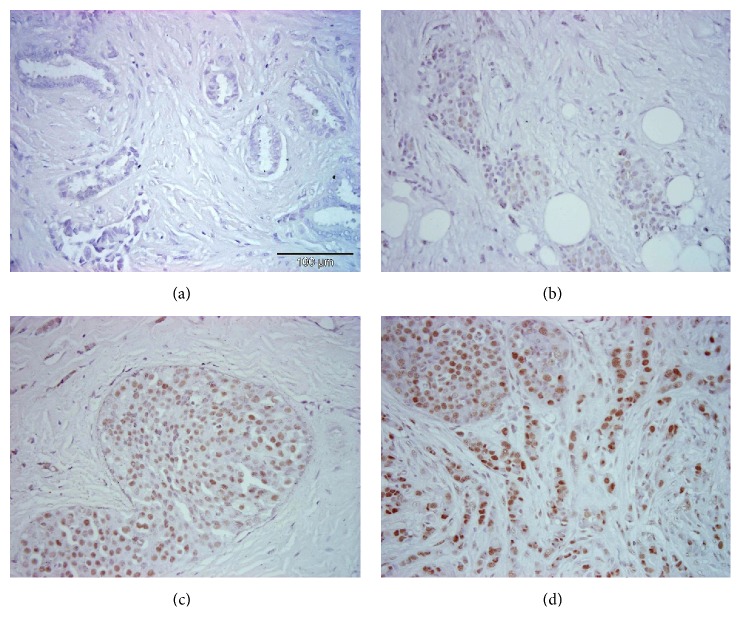
Examples of scored staining intensities: (a) negative control, (b) score 1, (c) score 2, and (d) score 3.

Survivin expression was analysed in 27 BC samples from 26 patients using immunohistochemical staining. Samples were scored between 1 and 3 based on survivin expression, taking into account the percentage of positive cells and the staining intensity (Figure 1). The numbers of samples exhibiting survivin expression with a score of 1 (weak), 2 (moderate), and 3 (strong) were 9 (33.34%), 11 (40.72%), and 7 (25.94%), respectively (Table 2). The majority of patients had nuclear survivin staining (92.6%), so we combined all the samples together for the statistical evaluation.

The high nuclear staining of survivin is different than most published data, where the nuclear staining is mostly lower than cytoplasmic [18, 28–31] and only rarely has been described as equal or higher than cytoplasmic [32, 33].

### 3.3. Association of Survivin Expression with Clinicopathological Parameters

**Figure 2:**
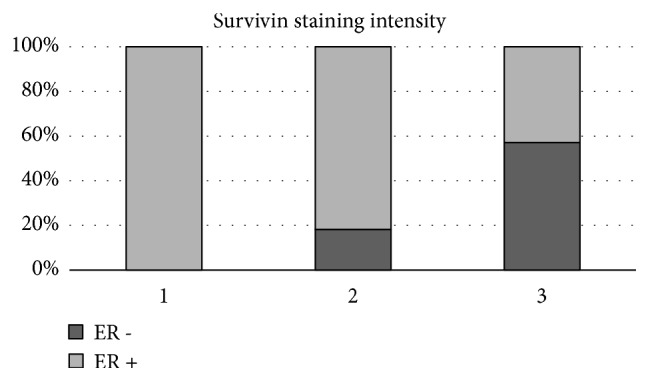
Association between survivin expression and ER status. Survivin expression was ranked weak, moderate, and strong, and percentage of ER negative and ER positive samples per group is shown.

Clinicopathological findings (age at diagnosis, time since operation, histological grade, tumour size, oestrogen and progesterone receptors, Her2 status, lymph node status, and Ki67 status) were compared with the immunohistochemical characterization of survivin expression. Since the majority of samples (22 of 27 samples, 84.6%) were ductal BC all samples were grouped together. The statistical analyses revealed a significant association between survivin expression and ER status. High survivin expression was significantly associated with negative ER status (p=0.007). No samples with low survivin expression (score 1), 18.2% samples with medium expression (score 2), and 57.1% of samples with high survivin expression (score 3) had negative ER status (Figure 2). Survivin expression was weakly associated with negative PR status (p=0.051). Furthermore, another statistically significant association was confirmed between survivin expression and Ki67 expression. High survivin expression was associated with positive Ki67 expression (p=0.032).

There is controversial data reported about association between high survivin expression and negative ER and/or PR status in BC. While some studies [34, 35] found the same association as here, other studies report these parameters as not significant [32, 36, 37]. The similar situation is with the association between high survivin expression and Ki67 expression. While some studies [38] showed the same results as here, others showed there was no significant association between survivin and Ki67 [31, 39].

### 3.4. *BIRC5* Polymorphisms in Breast Cancer Patients

Fifteen different polymorphisms were found in the constitutional DNA of 26 BC patients and 74 healthy controls.

The whole* BIRC5* gene coding region and selected promoter and 3'UTR regions were investigated for selected ten polymorphisms. One of the ten polymorphisms (c.-235G>A) was not found in BC patients. Polymorphisms and their frequencies are listed in Table 3.

**Figure 3:**
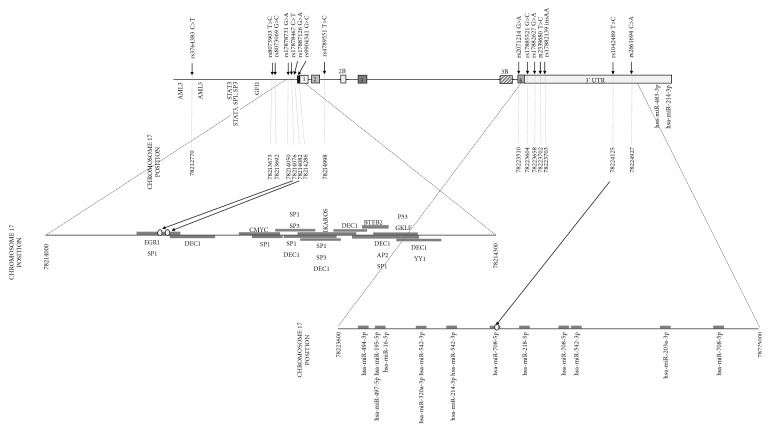
Structure of* BIRC5* gene with positions of analyzed polymorphisms. Positions of known binding sites for transcription factors and miRNA are shown according to TRANSFAC database [40]. Chromosome 17 positions are numbered according to human genome build GRCh38/hg38.

According to the TRANSFAC database (http://genexplain.com/transfac), promoter and 5'UTR region of* BIRC5* gene contain 29 binding sites for 14 different transcription factors. Chromosome positions of the detected polymorphisms revealed that polymorphisms rs17878467 (c.-241C>T) and rs17887126 (c.-235G>A) are located in the shared binding site of transcription factors EGR1 and SP1 (Figure 3). 3'UTR region also contains 16 binding sites for 11 different miRNAs, and only polymorphism rs1042489 (c.9809T>C) is located in the binding site of hsa-miR-708-5p (Figure 3) [40].

Fourteen different polymorphisms were found in BC samples while in controls one more polymorphism was present (c.-235G>A). The majority of detected polymorphisms were located either upstream or downstream of the coding region of the gene, and only one in the coding region. In addition to ten polymorphisms selected for this study, five more rare polymorphisms were found: one in the promoter (c.-267G>A), one in intron 2 (c.221+209T>C), and 3 in the 3'UTR (c. 9288G>C, c.9342G>A, and c.9387_9388insAA). There was no significant difference in genotype or allele frequencies between BC and control samples.

The observed allele frequencies for most of the polymorphisms are in accordance with previously observed frequencies for either global or European populations [15, 19, 20, 22, 41–43], or in NCBI database (http://www.ncbi.nlm.nih.gov), and only for c.221+209T>C the frequency was lower in this study than previously observed [41, 44]. c.-235G>A polymorphism found only in healthy controls is known to create a new “alternative” binding site for the transcription factor GATA-1 and is suggested to increase the BC aggressiveness [45]. Since most of the patients in this study are still alive, it is possible that this is a reason this polymorphism was not found in the patient group.

**Figure 4:**
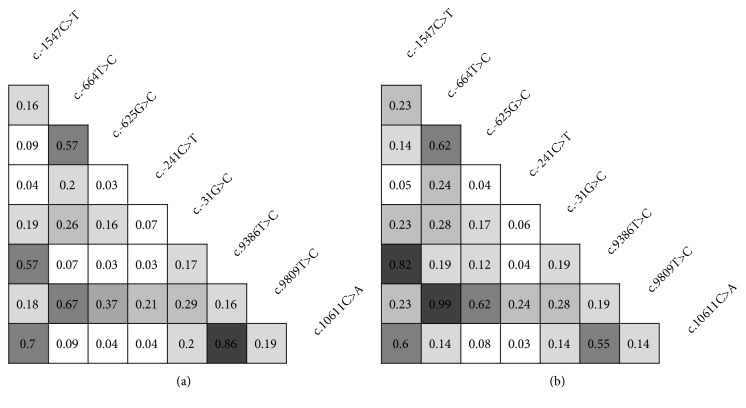
Pairwise linkage disequilibrium (LD) of eight* BIRC5* sequence variants with highest minor allele frequency. The location of each sequence variant along the* BIRC5* gene is relative to the real nucleotide position. The number in each diamond indicates the intensity of LD (r^2^) between respective pairs of sequence variants. The LD strength is also represented by shades of grey (0 [white] < r^2^ < 1 [black]). (a) Controls. (b) Cases.

Analysis showed there is a difference in number of polymorphisms in linkage disequilibrium (LD) between BC samples and controls. BC cases showed increased nonrandom association of alleles at multiple loci. In case of c.-644C>T and c.9809T>C, these two polymorphisms always appear in the same combination in all tested BC samples showing complete LD (r^2^=0.99) (Figure 4). Only one pair of polymorphisms, c.9386T>C and c.10611C>A, shows decreased association in BC samples compared to control samples.

LD between various* BIRC5* polymorphisms have often been reported in different populations: some observed here, but also others. LD between c.-1547C>T and c.9368 found here has been reported by Shi et al. [15], as were c.-1547C>T with c.10611C>A and c.9386T>C with c.10611C>A. The latter one was also reported by Pu et al. [46]. LD between c.-644T>C and c.-625G>C was reported twice, by Jang et al. [19] and Yang et al. [42], and between c.-625G>C and c.9809T>C once [47]. Interestingly, the polymorphisms in total LD, c.-644C>T and c.9809T>C, found in BC patients have not been described before.

### 3.5. Association of* BIRC5* Polymorphisms with Survivin Expression and Clinicopathological Parameters

**Figure 5:**
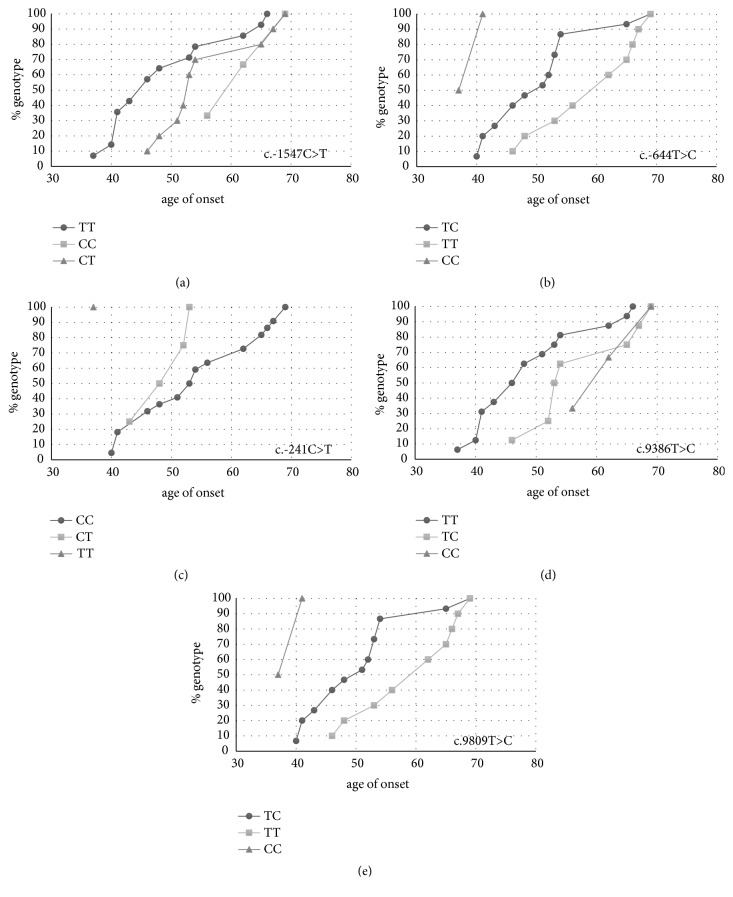
Association of age of onset with the significantly associated polymorphisms. (a) c.-1547C>T. (b) c.-644T>C. (c) c.-241C>T. (d) c.9386T>C. (e) c.9809T>C.

*BIRC5* polymorphisms were not associated with survivin expression, but several polymorphisms were found to be associated with age of onset. Genotype frequencies of polymorphisms c.-644T>C, c.9386T>C, and c.9809T>C were significantly associated with age of onset, while c.-1547C>T was weakly associated. Allele frequencies of these four, as well as SNP c.-241C>T, were also associated with age of onset (Table 4). When genotype frequencies were plotted against age of onset, a clear trend could be seen for the five polymorphisms (Figure 5) (the results are presented as in Han et al.'s study [4]).

For the c.-1547C>T polymorphism, the minor allele T was associated with earlier age of onset. The same trend is visible for the c.-644C>T allele and the c.9089C>T allele, where in both cases the minor C allele is associated with earlier age of onset. For the c.9386T>C allele, the major allele T is associated with earlier age of onset. For the final, c.-241C>T polymorphism, where the difference was significant only on allele but not on genotype frequencies, the minor allele T also seems to be associated with earlier age of onset but the number of samples in the TT group is too low to make this comparison.

Until now, only two studies showed association between* BIRC5* polymorphisms and age of onset. Han et al. [4] found significant connection of age of onset for the c.-1547A>G and less significantly c.-31G>C polymorphisms in ovarian cancer, while Hmeljak et al. [20] found that c.-241C>T was associated with the age of diagnosis of malignant pleural mesothelioma patients, but not with total survival. These results are comparable with our results in BC, where we show that the minor allele of the c.1547A>G polymorphism is also associated with age of onset, and in our case the results for the c.-241C>T are significant when comparing allele, but not for genotype frequencies. This discrepancy may be due to a small number of samples with the TT genotype (only one in our dataset). In BC, amplifications in* BIRC5* gene were also associated with the younger age of onset [48].

## 4. Conclusions

This was the first study investigating the possible role of* BIRC5* polymorphisms in breast cancer etiology conducted in Croatia. In our study no association of any of* BIRC5* polymorphism with BC or level of survivin expression was observed. High survivin expression was significantly associated with negative ER status. However, as many as five* BIRC5* polymorphisms were associated with the age of onset, a phenomenon which was previously observed only in ovarian cancer patients. Two of those five polymorphisms (c.-644T>C and c.9809T>C) were also shown to be in complete LD in breast cancer patients. We are aware that the sample size of this study was limited, but it should be noted that the allele frequencies for polymorphisms found were in accordance with previous reports, except for c.-235G>A, which was not present in BC samples. To assess the significance of* BIRC5* polymorphisms and survivin expression as predictive and prognostic biomarkers for BC further research with a larger sample size is needed.

## Figures and Tables

**Table 1:** Patient clinicopathological characteristics.

Characteristic	No. of patients (%)
Age at diagnosis (years)	

< 40	1 (3.8)
40-50	9 (34.6)
50-60	8 (30.8)
> 60	8 (30.8)

Years since operation	

5	8 (30.8)
10	15 (57.7)
15	3 (11.5)

Histological grade	

1	4 (14.8)
2	12 (44.4)
3	10 (37.0)
N.A.^*∗*^	1 (3.7)

Tumour size	

1 (≤ 2 cm)	20 (74.1)
2 (> 2 ≤ 5 cm)	6 (22.2)
3 (> 5 cm)	1 (3.7)

Oestrogen receptor status	

Positive	21 (77.8)
Negative	6 (22.2)

Progesterone receptor status	

Positive	16 (59.3)
Negative	11 (40.7

Her2 status	

Positive	2 (7.4)
Negative	22 (81.5)
N.A.	3 (11.1)

Lymph node status	

Positive	11 (40.7)
Negative	16 (59.3)

Pathologic Characteristics	

Invasive ductal carcinoma	22 (81.5)
Medullary carcinoma	2 (7.4)
Invasive lobular carcinoma	1 (3.7)
Mixed invasive ductal/lobular carcinoma	1 (3.7)
Papillary carcinoma	1 (3.7)

Ki67 status	

Positive	12 (44.4)
Negative	12 (44.4)
N.A.	3 (11.1)

*∗* Patient data not available.

**Table 2:** Levels and localization of survivin expression.

Characteristic	No. of samples (%)
Survivin immunoreactivity score	

weak	9 (33.3)
moderate	11 (40.7)
strong	7 (25.9)

Survivin cellular localization	

cytoplasmic	1 (3.7)
nuclear	25 (92.6)
cytoplasmic+nuclear	1 (3.7)

**Table 3:** Comparison of minor allele frequencies of *BIRC5* polymorphisms between breast cancer patients and healthy control samples. Nucleotide changes are named according to the literature.

gene region	SNP ID number	nucleotide change	minor allele frequency controls (n/N, %)*∗*	minor allele frequency BC samples (n/N, %)*∗*	p (for allele frequencies)
promoter	rs3764383	c.-1547C>T*∗∗*	37/148 (25.0)	16/52 (30.8)	0.466

promoter	rs8073903	c.-644T>C	49/148 (33.1)	18/52 (34.6)	0.865

promoter	rs8073069	c.-625G>C	33/148 (22.3)	13/52 (25.0)	0.704

promoter	rs17878731	c.-267G>A	1/148 (0.7)	1/52 (1.9)	0.453

promoter	rs17878467	c.-241C>T	16/148 (10.8)	6/52 (11.5)	1.000

promoter	rs17887126	c.-235G>A	2/148 (1.3)	0/52 (0)	

5'UTR	rs9904341	c.-31G>C	55/148 (37.2)	18/52 (34.6)	0.867

intron 2	rs4789551	c.221+209T>C	7/148 (4.7)	1/52 (1.9)	0.683

exon 4	rs2071214	c.9194G>A*∗∗∗*	5/148 (3.4)	1/52 (1.9)	1.000

3'UTR	rs17885521	c.9288G>C	3/148 (2.0)	1/52 (1.9)	1.000

3'UTR	rs17882627	c.9342G>A	2/148 (1.3)	2/52 (3.8)	0.278

3'UTR	rs2239680	c.9386T>C	34/148 (23.0)	14/52 (26.9)	0.575

3'UTR	rs17882139	c.9387_9388insAA	3/148 (2.0)	2/52 (3.8)	0.606

3'UTR	rs1042489	c.9809T>C	53/148 (35.8)	18/52 (34.6)	1.000

3'UTR	rs2661694	c.10611C>A	38/148 (25.7)	11/52 (21.1)	0.578

*∗* n – number of minor alleles, N – number of analyzed alleles

*∗∗*C is the minor allele

*∗∗∗*G is the minor allele.

**Table 4:** Association of genotype and allele frequencies of polymorphisms with age of onset.

polymorphism			mean±SD (years)	p-value
c.-1547C>T*∗*	genotype	CC	62.3±6.5	0.054
		CT	55.8±8.1	
		TT	49.0±10.1	
	allele	C	58.3±7.9	**0.011**
		T	50.9±9.8	

c.-644T>C	genotype	TT	59.4±8.2	**0.006**
		TC	50.7±8.6	
		CC	39.0±2.8	
	allele	T	55.8±9.2	**0.006**
		C	48.1±9.1	

c.-241C>T	genotype	CC	54.7±10.0	0.143
		CT	49.0±4.5	
		TT	37.0±0.0	
	allele	C	54.2±9.6	**0.029**
		T	45.0±7.1	

c.9386T>C	genotype	TT	49.1±9.3	**0.029**
		TC	57.4±8.4	
		CC	62.3±6.5	
	allele	T	50.8±9.6	**0.004**
		C	59.5±7.6	

c.9809T>C	genotype	TT	59.4±8.2	**0.006**
		TC	50.7±8.6	
		CC	39.0±2.8	
	allele	T	55.8±9.2	**0.006**
		C	48.1±9.1	

*∗* C is the minor allele.

## Data Availability

This is not applicable, since there are no big datasets connected with this article.

## Supplementary Materials

Supplementary Materials Supplementary Table 1: PCR primer sequences and cycling conditions.
