# Supplementary material for: Polymorphisms in Survivin (BIRC5 Gene) Are Associated with Age of Onset in Breast Cancer Patients
Source: J Oncol. 2019 Jul 28;2019:3483192. doi: 10.1155/2019/3483192 (PMC6699404; doi:10.1155/2019/3483192)
Supplement: Supplementary Materials — Supplementary Table 1: PCR primer sequences and cycling conditions. [file 3483192.f1.docx]

Supplementary table 1: PCR primer sequences and cycling conditions. Primer names of the fragments located in the regulatory regions are according to the major polymorphism present in the fragment.

| fragment name | primer sequence | Tm/°C | fragment size/bp |
| --- | --- | --- | --- |
| rs3764383 | F – GGAGGAGAGAAAGGGAGGAA  R – ACCTCAAGTGATCTGCCTGC | 66 | 209 |
| rs8073903 | F – CCCCTGACTCCAGAAGGTG  R – TCAGACAGGAGAGCTTTACAGG | 60 | 175 |
| rs17878467 | F – GATTACAGGCGTGAGCCACT  R – GTGTGCCGGGAGTTGTAGTC | 62 | 159 |
| *BIRC5* Exon 1 | F – GACTACAACTCCCGGCACAC  R – CCTCCAAGAAGGGCCAGT | 58 | 251 |
| *BIRC5* Exon 1a | F – AGCCCTTTCTCAAGGACCAC  R – CTCGATGGGGACAAAGCAG | 60 | 264 |
| *BIRC5* Exon 2 | F – CACTCACGAGCTGTGCTGTC  R – GAAGCAATGAGGGTGGAAAG | 62 | 250 |
| *BIRC5* Exon 2a | F – CGATGGGCTTTGTTTTGAAC  R – CAGGGTCTGCTGATGTATTCTG | 60 | 242 |
| *BIRC5* Exon 2B | F - CCCTTCTCTGCCCTTAATCC  R – TAGTGGAGACGGGGTTTCAC | 64 | 147 |
| *BIRC5* Exon 3 | F – AGAGGTGCCATATGGGAATG  R – CATTGAACAGGGTTTGAGCA | 62 | 342 |
| *BIRC5* Exon 3B | F – CCCTGGATTTGCTAATGTGA  R – AGCTCTGCTCTTAACCACTGC | 60 | 229 |
| *BIRC5* Exon 4 | F – CTGGGAAGCTCTGGTTTCAG  R – CTGGTGCCACTTTCAAGACA | 62 | 310 |
| rs1042489 | F – TGCATGACTTGTGTGTGATGA  R – CCGTTTCCCCAATGACTTAG | 60 | 161 |
| rs2661694 | F – TGTATCATCCGGGCTCCTT  R – ACAGAGGCTGGAGTGCATTT | 60 | 159 |
